
JOURNAL INFORMATION
==============================
NLM Title Abbreviation: ScientificWorldJournal
Journal ID: ScientificWorldJournal
NLM Journal ID: 101131163
Journal ID: TSWJ

The Scientific World Journal

ISSN: 2356-6140
EISSN: 1537-744X
Publisher: Wiley

ARTICLE INFORMATION
==============================
PMCID: PMC5917286
DOI: 10.1100/tsw.2006.401
Article ID: 689284
Article version: 1
Subjects: Publisher's Note

Publication of a Special Issue: Robotic Surgery in Urology

Redhead Claire
TheScientificWorld, Ltd., UK
Publication date: 2006
Electronic publication date: 2006 Sep 19
Volume: 6
First page: 2602
Last page: 2602
Copyright: Copyright © 2006 Claire Redhead.
Copyright year: 2006
License: This is an open access article distributed under the Creative Commons Attribution License, which permits unrestricted use, distribution, and reproduction in any medium, provided the original work is properly cited.
License URL: https://creativecommons.org/licenses/by/3.0/

==============================
Publisher's Note
Robotic Surgery in Urology
*TheScientificWorldJOURNAL*, (2006) 6, 2602
TSW Urology
ISSN 1537-744X; DOI 10.1100/tsw.2006.401

TheScientificWorldJOURNAL
www.thescientificworld.com

### Publication of a Special Issue: Robotic Surgery in Urology

Claire Redhead
*TheScientificWorld, Ltd., UK*

*Published September 19, 2006*

---

TheScientificWorldJOURNAL and TSW Urology are pleased to have published the special issue 'Robotic Surgery in Urology', a collection of peer-reviewed papers on a range of key topics in this field. Organized by Dr. William Steers (Hovey Dabney Professor and Chair, Department of Urology, University of Virginia School of Medicine), this special issue explores the rapidly escalating use of robotics in urological surgery and features papers on ranging from the economics and logistics of setting up a program to the technical details of robotic prostatectomy, cystectomy, pyeloplasty, and other pelvic surgeries.

Specific topics covered are:

- Pediatric Robotic-assisted Laparoscopy: A Description of the Principle Procedures
- Tips on Establishing a Robotics Program in an Academic Setting
- Update on Robotic Laparoscopic Radical Prostatectomy
- Robotic Assisted Prostatectomy: A review of Vattikuti Institute Prostatectomy (VIP). 1500 cases.
- Robot-assisted radical cystectomy in the management of bladder cancer
- Future directions of robotic surgery: Case study of athermal robotic technique of prostatectomy.
- Histopathologic outcomes of robotic radical prostatectomy

The Publisher wishes to thank the authors and reviewers, and especially acknowledges the contribution of Dr. Steers in bringing about the publication of this special issue.

**This article should be cited as follows:**

Redhead, C. (2006) Publication of special issue: robotics surgery in urology. *TSW Urology* **1(S1),** 73. DOI 10.1100/tswurol.2006.91.

©2006 with author.
